# Prioritizing Neighbourhood Amenities to Enhance Neighbourhood Satisfaction: A Case Study in Wuhan, China

**DOI:** 10.3390/ijerph20043528

**Published:** 2023-02-16

**Authors:** Qi Zhang, Zhenhua Zheng, Dezhi Kang, Ying Zhou, Yifeng Zhang, Xu Zhang

**Affiliations:** Wuhan Natural Resources Conservation and Utilization Center, Wuhan 430014, China

**Keywords:** amenities, perception, satisfaction, China, urban planning

## Abstract

In China, the improvement in amenities has been often criticized for not addressing the priorities of residents’ demand due to over-standardised, top–down practices and the misallocation of resources. Previous studies have investigated how people’s wellbeing or quality of life is associated with neighbourhood attributes. However, very few have researched how identifying and prioritizing the improvement in neighbourhood amenities could significantly enhance neighbourhood satisfaction. Therefore, this paper investigated the residents’ perception on the neighbourhood amenities in Wuhan, China, and explored the application of the Kano–IPA model for prioritizing the improvement in amenities in both commodity-housing and traditional danwei neighbourhoods. Firstly, total 5100 valid questionnaires were distributed through street face-to-face surveying to solicit the residents’ perceptions of the usage and satisfaction of amenities in different neighbourhoods. Then, various statistical techniques, including descriptive, logistical regression modelling were adopted to analyse the general characteristics and significant associations of amenities’ usage and demand. Lastly, an age-friendly strategy for the improvement in amenities in old neighbourhoods was proposed by referring to the widely applied Kano–IPA marketing model. The results showed that there is no significant difference in the usage frequency of amenities among different neighbourhoods. However, significant differences of associations between residents’ perception on amenities and neighbourhood satisfaction were identified among different groups of residents. To demonstrate prioritizing neighbourhood amenities in double-aging neighbourhoods, basic, excitement, and performance factors fitting age-friendly scenarios were determined and categorized. This research can provide a reference for allocating financial budgets and determining schedules to improve neighbourhood amenities. It also showcased the variances of residents’ demands and the provision of public goods among different neighbourhoods in urban China. Similar studies can be expected in addressing different scenarios that challenges emerged, such as suburban or resettled neighbourhoods where low-income residents generally live.

## 1. Introduction

A neighbourhood is the place where residents spend most of their lifetime; thus, neighbourhood environments are arguable associated with people’s wellbeing [1]. As essential attributes of the neighbourhood, neighbourhood amenities provide various services to support residents’ daily life. To what extent residents are satisfied with the neighbourhood amenities is commonly used to not only evaluate the quality of the provision of amenities, but also the quality of neighbourhood life [2,3]. Satisfaction with one’s neighbourhood is also a major component of overall life satisfaction [4]. Meanwhile, the provision of amenities is one of the key issues the developer and authorities have been working on to develop quality and liveable neighbourhoods. Thus, understanding how neighbourhood amenities is associated with residents’ satisfaction with their overall neighbourhood life is important for navigating neighbourhood development towards being more people-centric and enhancing neighbourhood satisfaction.

In China, emerging neighbourhood challenges derived from rapid urbanization and migration mobility, such as facility deterioration, social exclusion, inadequate open spaces, etc., have driven governments to revitalise the old neighbourhoods. To cope with the sharply decaying built environment, authorities and professionals have highly committed to improving the provision of amenities through urban renewal. According to international practices, evaluating the existing circumstances of neighbourhood development and illustrating the blueprint, particularly with the residents, are essential parts of neighbourhood planning [5]. Given that the prototype of urban planning has been macronarrative and top–down oriented for a long time in China, bottom–up neighbourhood planning has not drawn enough attention from both the authorities and the public. Thus, although neighbourhood planning in China is rapidly developing, the exploration of new paths and methods for neighbourhood planning is still at the inception stage. Recently, the ”neighbourhood life unit”, which emphasizes on the allocation of accessible amenities within walkable distance from the homes of residents, has been used as a new and official prototype to guideline neighbourhood planning nationwide. Additionally, public opinions towards neighbourhood development have been more respected and collected to enhance public participation. However, how residential perceptions and satisfaction can be concretely transformed into implications for people-centric planning is a common obstacle [6].

The associations between the people’s perceptions with amenities and neighbourhood satisfaction provide insight into ways to improve neighbourhood satisfaction. Previous studies have investigated the significant associations between neighbourhood satisfaction and various attributes, such as neighbourhood type, housing facilities, neighbourhood location, density, transportation facilities, accessibility to amenities, safety and social environment, etc. [7,8,9,10,11]. Moreover, the dominant contributors were identified by evaluating the relative importance of and variation in neighbourhood attributes [12,13]. Furthermore, the asymmetric relationships between service attributes and overall satisfaction can be ascertained through a satisfaction study [14]. As popular techniques in the field of customer satisfaction, the importance–performance analysis (IPA) and Kano model are used to assess service quality by identifying their associations with residential satisfaction. Based on the assessment, strategies can be proposed for prioritizing the service improvement. However, cross-disciplinary applications of the model in urban studies are limited to transport services, pedestrian satisfaction, and noise and public open space applications [14,15]. Particularly, the application of this model for prioritizing neighbourhood amenities to enhance neighbourhood satisfaction is rare.

Thus, this case study in Wuhan, China, aims to deepen the understanding of associations between attributes of amenities and overall neighbourhood satisfaction, based on which the priorities for the improvement in amenities can be identified to improve neighbourhood satisfaction. Through adopting the IPA–Kano model and logistical regression, the significant associations among various amenities and overall neigbourhood at different performance level can be identified and compared. In this regard, identifying causality is not the focus of this study. The term “neighbourhood satisfaction” is used to represent the “residential satisfaction with neighbourhood life” in this study. The paper is a solid extension of the authors’ previously published conference paper [16], which preliminarily introduced the case study. It is organized into seven sections. The second section reviews the relevant literature about neighbourhood satisfaction, amenities, and quality of life, together with importance–performance analysis and three-factor theory of satisfaction. The third section presents the methodology adopted, including conceptual framework, case selection, data collection, and analysis. The fourth section briefly describes the results of questionnaire surveys and summarizes the main characteristics of residents’ perception and usage with amenities. Fifthly, the application of the IPA–Kano model in prioritizing neighbourhood amenities to enhance elderly satisfaction in old danwei neighbourhood is presented. Lastly, the pattern of amenity usage and the problems of amenity provision are discussed and several policy implications proposed prior to the conclusions of the paper.

## 2. Literature Review

### 2.1. Neighbourhood Satisfaction, Amenities, and Quality of Life

Satisfaction with life largely depends on the extent to which individuals’ needs are fulfilled according to need theory [17]. Thus, satisfaction with the neighbourhood is significantly associated with the degree of fulfilment with residents’ aspiration on the services provided by the neighbourhood. Urban studies define the residential satisfaction with neighbourhood life as the resident’s subjective assessment of one’s life conditions based on the comparison among aspirations and achievements for various aspects of the neighbourhood they are living in. Both physical and social attributes contribute to residents’ satisfaction with neighbourhood life. On one hand, since neighbourhood environment is a concentration of the attributes affecting one’s life, such as the quality of amenities, the access to public transport, the adequacy of open spaces, etc., the objective and physical attributes are crucial to the quality of life [18]. Neighbourhood amenities are important parts of physical attributes, which normally include shopping, municipal service, education, medical, transport, fitness, open space, entertainment, and administrative service facilities. On the other hand, the multiple discrepancies theory emphasized the effect of social comparison in explaining the variance in residential satisfaction. It argues that life satisfaction is “inversely related to the degree of discrepancy from multiple standards, including what one wants, what one has had in the past, and what relevant others have” [17]. Thus, neighbourhood satisfaction is an integrated and multilayered indicator system, and an accumulative consequence of personal feelings.

Additionally, the importance of neighbourhood satisfaction lies on its mediating effect between neighbourhood attributes and residents’ quality of life [19]. Quality of life refers to a sense of wellbeing in a person’s life and the ability to live successfully and peacefully in the living environment [20], or how the person’s sense of life changes entirely to achieve a better future and develop an understanding of how they live within a culture and system [21]. Campbell argues that the residential environment may affect life satisfaction indirectly through individuals’ perceptions and evaluations of such an environment from the perspective of environment psychology [4]. Given its mediative role between objective attributes and subjective quality of life, neighbourhood satisfaction was used as one of the indicators to evaluate the quality of planning and development of the neighbourhood in urban planning practices [22]. It was justified that the priority of providing any of the facilities should be helping by satisfying the involved residents in the neighbourhood [23].

However, previous studies determined the complexity of the mechanism of how neighbourhood amenities affect residents’ subjective wellbeing. Some studies showed that the impact is limited in certain scenarios. Liu et al. (2017) investigated the influence of the residential environment on migrants’ subjective wellbeing in Guangzhou, China [24]. No significant association between neighbourhood amenities and migrants’ life satisfaction was found in their study. Dong and Qin (2017) argued that the impact of the neighbourhood environment on the subjective wellbeing is obvious, but only at a minor level in Beijing, China [25]. Comparatively, they determined that safety, residential convenience, and transit accessibility were the most influential attributes. On the contrary, Lee proofed that neighbourhood facilities had the most significant influence on quality of life in Gyeonggi, South Korea [26]. In addition, Ibem, Opoko, and Aduwo demonstrated the influence that residents’ satisfaction with services and infrastructure had on neighbourhood satisfaction with public housing in Ogun State, Nigeria [27]. Thus, the degree and logical link of how various neighbourhood amenities affect the residents’ wellbeing tends to be further explored.

### 2.2. The Importance–Performance Analysis and Three-Factor Theory of Satisfaction

How can the associations between amenities and neighbourhood satisfaction be understood and applied to improve the residents’ neighbourhood satisfaction through urban renewal is one of the key issues faced by urban planners. To address a similar scenario, the importance–performance analysis (IPA) and Kano model have been widely adopted for customer satisfaction evaluation in the marketing realm [28].

**Figure 1:**
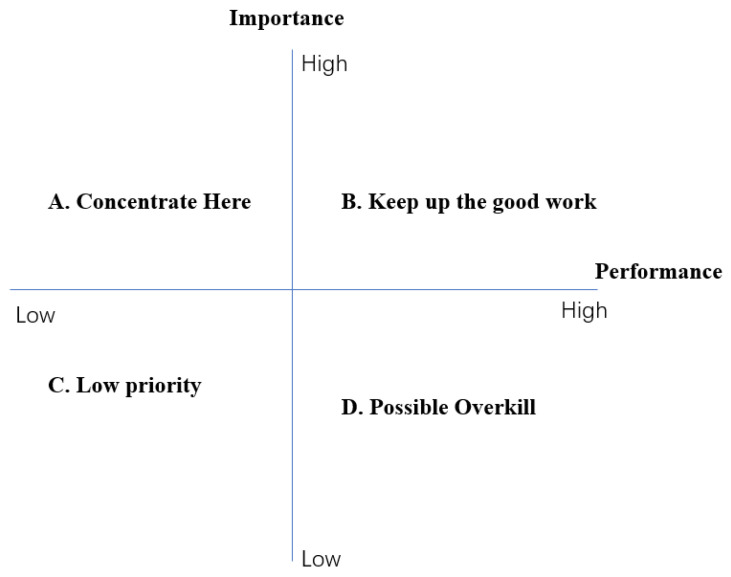
Importance–performance analysis grid.

As shown in Figure 1, the importance–performance analysis grid uses four quadrants divided by a vertical axis (importance) and horizontal axis (performance) to define all the factors of service and determine the priorities. For example, a factor would be categorized as “low priority” if its performance was “low” and, meanwhile, its importance was “low”. Two hypothesises are set for the model of IPA. Firstly, the importance and performance of all attributes are independent. Secondly, there is a linear and symmetric relationship between attribute performance and overall performance. Hereby, the four groups of attributes can be categorized as different levels of priorities. However, the disadvantages of the IPA were determined by some scholars who proved that asymmetric relationships existed between service attributes and overall performance [29,30].

**Figure 2:**
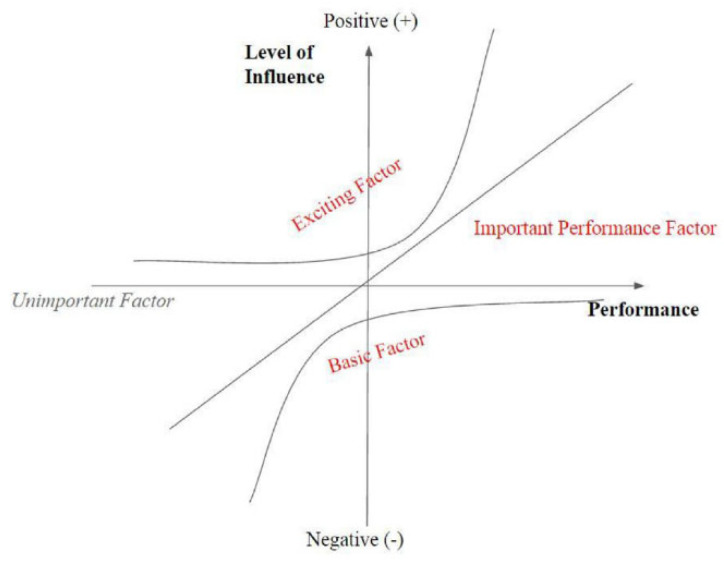
The method of categorization of three factors based on influence and performance [31].

To cope with the disadvantage of IPA in hypothesis, Kano et al. (1984) [31] originally proposed a three-factor theory that was adapted by subsequent studies on customer satisfaction [32,33,34]. As shown in Figure 2, the three-factor theory categorizes all the attributes based on their different levels of importance to overall satisfaction. The three groups of factors are basic factors, performance factors, and excitement factors. Basic factors are those attributes which do not increase overall satisfaction when they perform well but significantly decrease overall satisfaction when they perform poorly. On the contrary, exciting factors significantly improve the overall satisfaction only when they perform well. However, they do not significantly influence the overall satisfaction when they do not perform well. Comparatively, performance factors have a linear and symmetric relationship with overall satisfaction. Therefore, they significantly increase the overall satisfaction when they perform well while significantly decrease the overall satisfaction when they perform poorly.

To date, even though the three-factor theory has been widely applied in the realm of market studies, its application in urban studies is yet to be explored. Yin et al. (2016) adopted the three-factor theory to ascertain the environmental factors that affect residential satisfaction in China [35]. Dong et al. (2019) integrated the method with gradient-boosting decision trees to examine pedestrian satisfaction in gated communities [14]. Fewer studies have adopted three-factor theory in prioritizing the amenity improvements [36]. Given the limitations of resources and time available to governmental authorities, this research gap became more urgent in urban China, particularly in urban old districts where the built environment is rapidly decaying [37].

## 3. Methodology

### 3.1. Conceptual Framework

**Figure 3:**
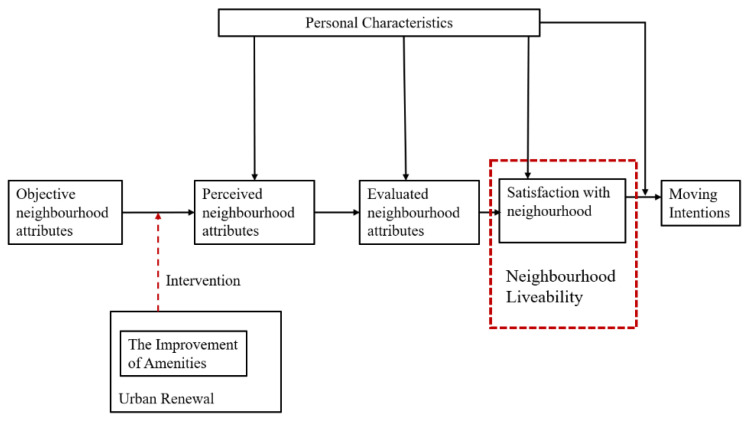
The framework for affecting neighbourhood satisfaction by improving neighbourhood amenities (adapted from Campbell et al. (1976) [2]; Marans and Rodgers (1975) [38], and Low et al. (2018) [39]).

Figure 3 shows how the objective attributes affect residents’ satisfaction with the neighbourhood and their intentions to move. Then, personal characteristics impose wide effects on the residents’ subjective perceptions, evaluations, and satisfaction from the perspective of environmental psychology. As an intervention into the objective attributes, the improvement in amenities through urban renewal may affect how the residents perceive the neighbourhood attributes, in which their neighbourhood satisfaction may change accordingly. In this research, variations in the impact of different amenities on the overall neighbourhood satisfaction were investigated.

### 3.2. Study Area and Case Selection

**Figure 4:**
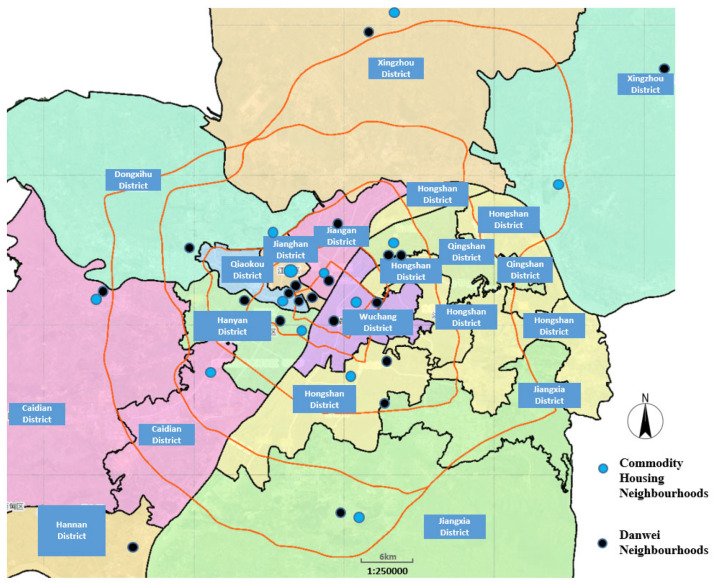
The location of all the 34 selected neighbourhoods in Wuhan.

Wuhan is the biggest megacity in central China and a good example that represents the urban transformation under significant institutional and economic transitions [40]. Danwei is a compound of employment that provides both working stations and living accommodations allocated to the employees. Prior to the late 1990s, danwei compounds provided most housing units in society. Additionally, it provided comprehensive and exclusive supportive facilities and services, including shops, parks, medical care, and educational facilities, as an employment benefit. Given that danwei and commodity housing are two major types of neighbourhoods in transitional China [41], these two categories were used to make the case selection from all 13 districts within Wuhan’s municipal boundary. Finally, a total of 34 neighbourhoods, including 13 commodity-housing neighbourhoods and 21 danwei neighbourhoods, were finalised for delivering questionnaire surveying (Figure 4).

### 3.3. Data Collection

The questionnaire survey was adopted to elicit both attitudinal data and demographic data. The types of neighbourhood amenities included in the questionnaires were defined based on the national “Planning and Design Standards of Urban Residential Area” of China. The six finalised categories of neighbourhood amenities in this study include: administrative and management; education; healthcare; service and business; civic and transportation; and public, cultural, and sport spaces. Furthermore, there are different numbers of specific amenities under each category (Table 1). Additionally, a Likert scale was adopted to formulate the choices for respondents to indicate their satisfaction with the corresponding amenity, where 5 represents strongly agree and 1 represents strongly disagree. The full version of the questionnaire survey (in Chinese) is presented in Appendix A.

In cross-sectional research, a sampling strategy that focuses on attaining small random samples with high response rates is considered more valuable than achieving large random samples with low response rates. Owing to the available resource and time restriction, high response rates are another focus of questionnaire collection rather than purposely increasing the sampling size in this research. Based on the determination considerations of sample size of previous survey study [42], 119 was determined as minimum returned sample size for a population size ranging from 4000 to 10,000 (margin of error = 0.05). Thus, given the population of one neighbourhood ranges from 5000 to 10,000 in Wuhan and the restricted research funding, the sampling size of each selected sample of neighbourhood was determined by the authors as 150. Accordingly, random sampling and face-to-face interviews were adopted in all 34 selected neighbourhoods, and the approached respondents were filtered by the trained interviewers based on the following selection criteria:

(1) Aged 18 or above;
(2) Permanent resident who is now living in the studied neighbourhood, excluding administrative staff or businessman on the street.

The whole interviewing process was conducted during November 2020. The interviewers first randomly approached the residents during the daytime and then asked the selected interviewees to mark their frequently used amenities and tick the corresponding option precisely representing their perceptions. For each of the selected neighbourhoods, 150 valid questionnaires were secured.

### 3.4. Data Analysis

Owing to the encountered objective and personal issues, parts of the distributed surveys were not validly finished. To secure the validity of the data obtained, questionnaires with any missing items were regarded as invalid and no longer processed in the data analysis.

Firstly, the content of collected questionnaire surveys were coded and transformed into numerical data. Then, the encoded data were tested by SPSS via reliability analysis. The results show that Cronbach alpha was 0.839 (larger than 0.8), which indicates a high level of reliability of the data obtained. Additionally, the result of a multicollinearity test showed that no independent variable was found with a tolerance smaller than 0.2, variance inflation factor (VIF) larger than 10, or eigenvalue is equal to 0. Therefore, there no multicollinearity existed among all the attitudinal variables, which were the independent variables in the model of this study.

Secondly, a descriptive analysis was conducted to establish the general circumstances regarding the residents’ perceptions of amenity provisions. Mean score ranking was adopted to examine the relative significance of individual factors. The Mann–Whitney U test was adopted to investigate whether there is a statistically significant difference between the means in two unrelated groups.

Thirdly, to prioritize the improvement in neighbourhood amenities in specific scenarios, the IPA–Kano model was used to identify and categorize the basic, performance, and exciting factors. By integrating the three groups of factors and their actual performances in Section 5, the study was able to identify the priorities for the provision of amenities to enhance the elderly satisfaction concerning neighbourhood life in an old danwei neighbourhood.

SPSS version 22.0 was used for conducting ordinal logistic regression modelling. Ordinal logistic regression is a statistical analysis method that can be used to model the relationship between an ordinal response variable and one explanatory variable. Based on the results, the basic, performance, and exciting factors can be identified and classified. In the logistical regression, the value of 3, which is in the middle of 1 to 5 of Likert Scale, was set as the reference value to test whether the good (value of 4 or 5) or poor (value of 1 or 2) performance of independent variables are significantly associated with the level of dependent variables. The dataset of variables whose value was 3 and 5 were interchangeably coded during the modelling. This ensured those groups whose independent variable was 3 were set as the reference groups. Additionally, the socioeconomic variables (Table 2) were regarded as controlled variables and included in the model. The p-value of the parallel-line hypothesis testing of all the generated models was larger than 0.05. Thus, the H0 hypothesis could not be rejected, which implied that ordinal logistic regressions were statistically suitable for data analysis. The correlation function of ordinal logistic regression is shown below:

$$Y^{\prime }=\mathrm{logit}\;[\pi (X)]=\mathrm{In}\frac{\pi (X)}{1-\pi (X)}=\alpha +\beta_{1}X1_{\;}+\beta_{2}X2_{\;}+\cdots +\beta_{p}X_{p}$$

Generally the mean value of overall satisfaction with neighbourhood life is 3.44, which shows that the overall level of satisfaction with neighbourhood life in Wuhan is at a medium level and there is not complete satisfaction. Specifically, the proportion of respondents who indicated either “satisfaction” or “high satisfaction” is 56.58%, which is below 60%. This implies that there is still room to improve the quality of neighbourhood life in Wuhan.

## 4. Results of Descriptive Analysis

### 4.1. Respondents’ Socioeconomic Characteristics

In total, 5259 surveys were distributed to selected respondents at various neighbourhoods. Through the validity check, the final number of valid questionnaires filled out was 5100, with 150 from each neighbourhood. Therefore, the valid rate of collected samples of questionnaires is 97.00%. The socioeconomic characteristics of the respondents are shown in Table 2. This set of data was reported in the authors’ previous conference paper [16], which demonstrated the initial results of the study.

### 4.2. Respondents’ Usage, Satisfaction, and Demands for Amenities

In this study, “the rate of frequent usage” of any specific amenity was defined as the proportion of respondents who ticked the corresponding amenity as a frequently used one (question No. 15 in Appendix A). Then “frequently used amenities” was defined as “over 50% of the respondents indicated they frequently used this amenity”. The results shows that the amenities whose rate of frequent usage is higher than 50% include supermarkets and convenience stores, fresh markets, restaurants, delivery points, subway stations, bus stations, and neighbourhood parks. In general, the amenities of daily life and business services, commuting-related, and public open spaces own higher rate of frequent usage than the others. Furthermore, most of these frequently-used amenities are those facilities within a ten-minute walking distance specified in the national standard of urban residential area. In comparison, the amenities whose rate of usage are below 5% include rehabilitation centres, elderly service centres, healthcare centres, police stations, bookstores, committee offices of property owners, fitness centres, private education institutes, and laundries. The lower rate of usage of laundries, private educational institutes, elderly care centres, etc. is probably due to either the exclusive usage from elderly, sports lovers, homemakers, etc., or the deficiency of current provision.

**Figure 5:**
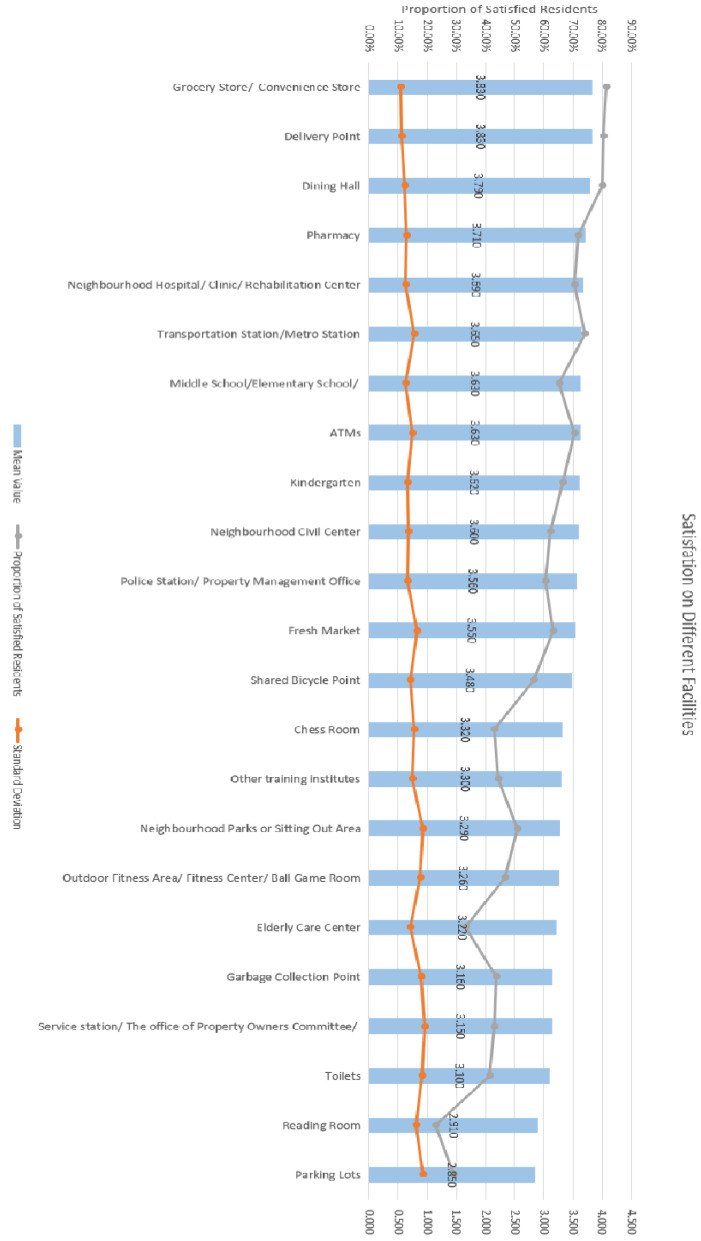
The mean values and variations in residential satisfaction for various amenities and proportion of satisfied respondents.

Additionally, concerning the mean value for all individual amenities (shown in Figure 5), the six amenities with the top-ranking mean values are shopping stores, delivery points, restaurants, pharmacies, medical care service centres, and bus stations. Comparatively, the last six amenities that have the lowest mean values are parking lots, reading rooms, public toilets, property management offices, garbage collection points, and elderly care centres (in ascending order). The proportion of satisfied respondents out of all respondents and the mean values and variations in residential satisfaction for various amenities are also illustrated in Figure 5.

**Figure 6:**
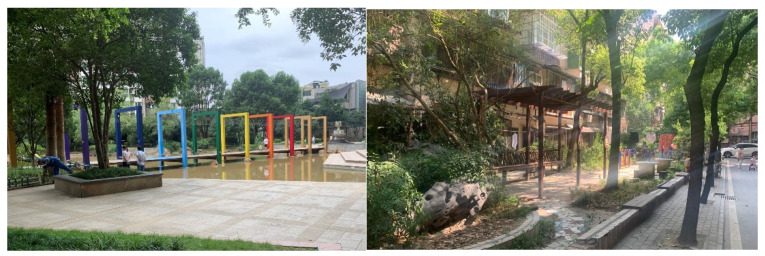
Public open spaces in a commodity-housing neighbourhood and old danwei neighbourhood.

Furthermore, amenities that are marked by the respondents as those “to be urgently improved” are public toilets, garbage collection points, outdoor activity areas, parking lots, pocket parks, elderly activity rooms, fresh markets, property management offices, etc. (in descending order) (Figure 6). Other than fresh markets, all the amenities perceived as “to be urgently improved” are also those with the lowest mean values of respondents’ satisfaction. Thus, these lower-graded amenities are highly desired by the respondents. The results show that the amenities with obvious NIMBY (not-in-my-backyard) effect significantly dissatisfy the respondents. It suggests that the authorities should substantially ensure the newly provisioned or renewal of these amenities to be people-centric rather than simply top–down procedure.

### 4.3. Higher Level of Satisfaction with Amenities in Commodity-Housing Neighbourhoods Than in Danwei Neighbourhoods

**Figure 7:**
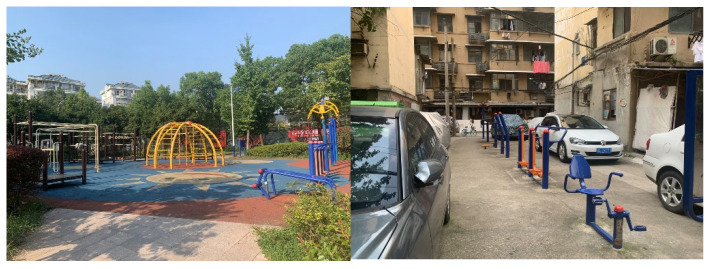
The fitness area in a commodity-housing neighbourhood (**left**) and a danwei neighbourhood (**right**).

The results of Mann–Whitney U tests showed no significant differences in amenity usage characteristics between traditional danwei neighbourhoods and commodity-housing neighbourhoods; however, the amenity satisfaction level of commodity-housing neighbourhood is generally and significantly higher than that of the traditional danwei neighbourhood. Out of the total twenty-three types of amenities, there are twenty whose mean value of satisfaction level is higher in commodity-housing neighbourhoods. On the contrary, restaurants, healthcare centres, and public toilets had a higher mean value of satisfaction in traditional danwei neighbourhoods. Thus, this proved that the commodity-housing neighbourhood can provide more satisfactory amenities to their residents than the danwei neighbourhood (Figure 7).

### 4.4. Frequently Used and Desirable Amenities for Different Age Groups

There are variances in amenity usage between the nonelderly and elderly groups. For the sum of the types of frequently used amenities (larger than 50%), there are a total of seven for the nonelderly group, whereas there are only three for the elderly group (Table 3). This demonstrates that the nonelderly group exhibits higher demand and stronger capability in using neighbourhood amenities. Individually, however, there are six types of amenities where the percentage of elderly frequent users is significantly larger than that for nonelderly frequent users. Furthermore, this shows that amenities frequently used by the elderly were mainly concentrated around certain specific amenities. It implies higher sensitivity by the elderly regarding fresh markets, neighbourhood parks, pharmacies, outdoor fitness areas, healthcare centres, and neighbourhood service stations.

Therefore, the results suggest that the elderly group’s quality of life has a higher degree of dependence on neighbourhood amenities. This demonstration can be supported by the results of the Spearman correlation analysis, which show that there are more types of amenities that have a higher impact (the correlation coefficient is larger than 0.3) on the elderly neighbourhood satisfaction than on the nonelderly groups. For the elderly group, the correlation coefficient between their overall neighbourhood satisfaction and elderly service centres, property management offices, fitness areas, reading rooms, neighbourhood squares, neighbourhood service centres are all larger than 0.3. Comparatively, the amenities whose correlation coefficient with overall neighbourhood satisfaction is larger than 0.3 only include bus stations and property management offices for nonelderly groups.

## 5. Prioritizing Neighbourhood Amenities to Enhance Elderly Satisfaction in Old Danwei Neighbourhoods: Application of the IPA–Kano Model

In China, the neighbourhoods built prior to 2000 are defined as “old neighbourhood” in the national project of “the renovation of old neighbourhood”. Owing to an early build-up time, limited space, and decaying built environments, the improvement in amenities is urgent in old danwei neighbourhoods. Both the increasing ageing population and buildings highlight the importance of improving amenities for fostering elder-friendly built environments. Thus, as a means to demonstrate how to prioritize improvements by applying the IPA–Kano model, this research hereby selected the elderly respondents living in old danwei neighbourhoods as an example.

By sorting all the respondents living in old danwei neighbourhoods and aged over 60 years, we were able to shortlist 482 out of the total 5100 questionnaires for modelling. Then, the results of the regression analysis demonstrated that there are 20 out of 23 factors significantly associated with overall neighbourhood satisfaction (Appendix B). By referring to the methods of the IPA–Kano model introduced in Section 3.4, the three factors and amenity performance were identified to propose the matrix. Based on the significant factors given in Appendix B and the performance of each variable (the mean value of satisfaction) in Table 4, the 20 variables were categorized into 9 specific groups with reference to the matrix, as shown in Table 5.

Based on the category of the matrix in Table 5 and the principles of priority identification in the model, the priorities of improvements enhancing elderly satisfaction in old danwei neighbourhoods can be proposed as follows. The top priorities should be given to those categorized as basic factors with poor performance, which include reading rooms and elderly service stations. Secondly, the amenities that should be then improved are those categorized as important performance factors with poor performance, which include property management centres, garbage collection points, neighbourhood squares, and parking lots. The third priority should be given to those categorized as basic factors with moderate performance. Other administrative facilities and service stations are those in point. Furthermore, the amenities categorized as important performance factors with moderate performance, including privately owned education and training centres along with chess rooms, should be subsequently improved. Lastly, those amenities categorized as exciting factors with either poor or moderate performance should be considered as the lowest priorities for improvement. These include fresh market, bank branches, kindergartens, public toilets, and neighbourhood service. Considering the scarce available space and weak accessibility due to elderly walking capabilities, it is recommended that the lacking amenities should be collectively allocated within an elderly walkable distance. Additionally, the results of correlation analysis and regression model show that gender significantly associate with the elderly overall satisfaction with the neighbourhood. Female elderly exhibit higher satisfaction than male elderly.

## 6. Discussion and Policy Implications

Following the objectives of the study, the general residents’ perceptions and usage of neighbourhood amenities, together with other socioeconomic characteristics, are discussed based on the descriptive analysis. Then, the variance in residents’ satisfaction and identified problems are further discussed to demonstrate the challenges and opportunities in the context of Wuhan. Lastly, several policy implications for planning and policy-making are proposed.

### 6.1. Pattern of Amenity Usage

**Figure 8:**
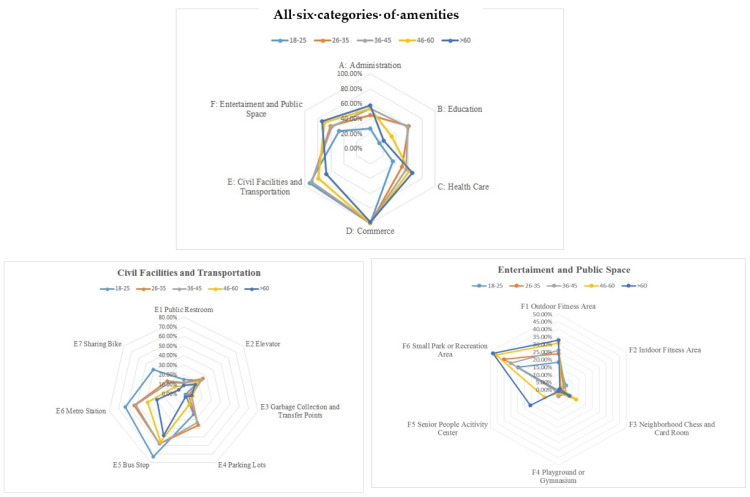
The percentage of frequent users in different age groups.

The results reveal that frequently used amenities include not only convenient services and transportation for commuting, but also delivery points and public spaces. This implies a change in lifestyles at a neighbourhood level. Previously, residents usually walked to the nearby groceries, supermarkets, fresh markets, or shopping malls to purchase daily items. Now, the demand for online shopping and contactless delivery is constantly increasing. As the lingering pandemic has hindered social interactions, accessible and efficient delivery points have become an essential amenity for every neighbourhood. Furthermore, the high frequency of usage of public open spaces demonstrates the important role of these spaces in residential daily routines. Particularly, the social wellbeing of the elderly and active ageing is highly and positively associated with public open spaces [43]. Figure 8 shows the percentage of frequent users of various amenities in different age groups. Accordingly, the elderly group shows a higher percentage of frequent users of administration spaces, healthcare facilities, and entertainment and public open spaces, and a lower percentage of users of civil, transportation, and educational facilities. This supports the findings from previous studies that explained how the elderly have a decreasing demand for basic materials, looking after others, and social networks, and an increasing demand for health and medical care as they become older [44].

Additionally, the elderly group accounts for the largest proportion (60.1%) of all respondents who chose “walking” as their major daily commuting method. Considering that only 15% of all respondents are elderly with poorer mobility and a concentrated number of frequently used amenities, the study’s findings support that residents’ dependence on and sensitivity for walking-accessible amenities becomes higher as they age [45]. Given the rapid ageing pace in China, developing age-friendly built environments in neighbourhoods should be urgently emphasized to meet the enormous demand for physical and psychological activities for the elderly.

### 6.2. Problems of the Provision of Amenities in Commodity-Housing and Danwei Neighbourhoods

Generally, by comparing the neighbourhood development patterns, commodity-housing neighbourhoods can provide more satisfactory amenities than danwei neighbourhoods. Through the statistical analysis and field observations, it was discovered that the improvement in amenities in old danwei neighbourhoods is much more urgent and challenging compared with that in new commodity-housing neighbourhoods. This is due to the significant housing reform that caused a switch from danwei compounds to commodity housing, and which improved the quality of provided neighbourhood amenities during the past few decades.

**Figure 9:**
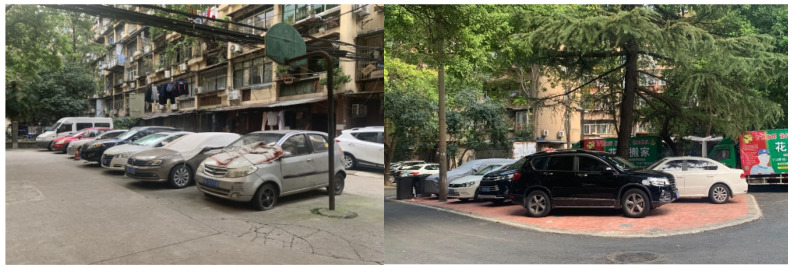
Parking lots occupy public spaces in Libei and Huaqiao neighbourhoods.

To date, given that old danwei neighbourhoods still accommodate a large population in the context of urban China, the improvement in residents’ quality of life is more vital and urgent in danwei neighbourhoods. Several outstanding problems in old danwei neighbourhoods were identified. The first is the spatial conflict between parking lots and open spaces (Figure 9). Owing to the increasing number of private cars and limited usable space within the neighbourhood, it was commonly found that extra parking lots were dispossessing the public open spaces. Secondly, the scarcity of elderly care centres and public toilets is another problem. Given the significantly increasing elderly population, the demand for ageing in place is rapidly increasing. The existing provisions of elderly care centres and public toilets are either deficient or unsatisfactory. This is mainly attributed to the lower planning standard when the neighbourhood was built. No exclusive space was reserved for the use of elderly care centres and public toilets at the time, and now, there is no usable space to be utilized. To supplement the deficiency, specific planning for elderly care centres and public toilets was already established years ago in Wuhan. The results of this study revealed that the implementation of this planning did not meet the residents’ demands for elderly care centres and public toilets.

**Figure 10:**
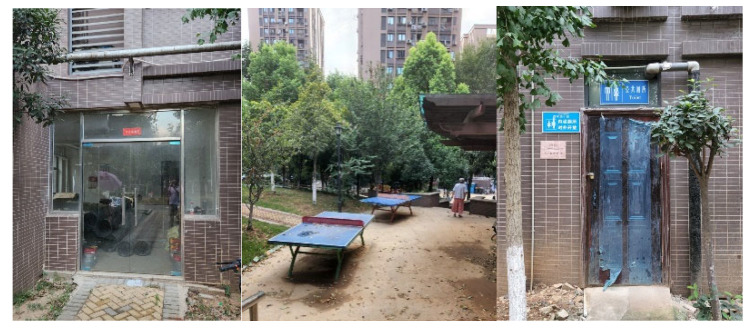
The vacant or blocked elderly activity room, toilets, and ping-pong tables in Qingheju neighbourhood.

Despite residents’ higher satisfaction with the provided amenities, some of the investigated commodity-housing neighbourhoods still face problems. Some amenities were identified as “vacant” in the field observations and interviews. For instance, the elderly activity room, public toilets, and ping-pong tables were found to be vacant or blocked for a long time in the Qingheju neighbourhood, which was built in 2015 (Figure 10). This is mainly attributed to management issues. Although these amenities were built together with the residential buildings by the developers, the subsequent clarification and transfer of property rights and the management mechanism remain unclear. Basically, the property rights of the amenities belong to all property owners. However, it usually takes a couple of years for the newly moved-in residents to elect members of an owners’ committee. Thus, prior to that, there are no legislative delegates to address the property rights and management issues regarding the amenities.

### 6.3. Policy Implications

Generally, the diversification and dynamics of residents’ demands for neighbourhood-based services nowadays require a more effective, diverse, and flexible system of amenities. This challenges the improvements in amenities because supplying amenities is a systematic project, which requires not only spatial planning and allocation, but also management and operations. In this sense, simply planning an adequate quantity of amenities is not enough to provide quality and sustainable services. The amenities are likely to be left vacant if the subsequent mechanism of operation is not well designed. To some extent, the disjunction and fragmentation of the planning, construction, and operation phases during the lifecycle process of the provision of amenities eventually hinder the quality of services provided. Thus, the government should improve the coordinative mechanism among different departments and enhance the systematic arrangements for the provision of amenities from initial planning to their long-term operation.

Currently, urban renewal has obtained strong governmental, financial, and human resource support. It provides a good opportunity to improve the supportive amenities and revitalise the built environment in decaying neighbourhoods. Compared with city-level public facilities, the importance of neighbourhood amenities that are within a 15-min walking distance was highlighted in this study. In old neighbourhoods, this study suggests that attention should be given to enhancing the quality rather than the quantity of existing amenities, given that the usable space is rare for allocating additional amenities. Other than spatial solutions, the improvement in identified problematic amenities also requires operation, legislation, and other policy-making decisions. For instance, the Wuhan municipal government has regulated that public toilets must be built in large-size neighbourhoods. However, the regulation department has not precisely defined the meaning of “large size”. Moreover, this regulation is just a local regulation that does not have a penalty provision. Thus, the regulation department of public toilets should work together with the legislative authorities to improve the regulations or ordinances that are not directly enforceable.

Next, engaging market entities as supplying systems for the neighbourhood amenities is critical for developing sustainable and liveable neighbourhoods. This study uncovered that the amenities provided by market entities, such as groceries, supermarkets, canteens, express delivery services, banks, and pharmacies, have a higher satisfaction among residents than those provided by the government. Particularly, the express delivery service in commodity-housing neighbourhoods has the highest level of residents’ satisfaction. This proves that the market-driven provision of amenities is highly efficient and effective. Thus, engaging market entities as the provision systems for neighbourhood amenities is essential to improve the quality of service and ease governmental financial pressure. Thus, clearly listing the type of amenities where market access is allowed can be considered to encourage the participation of the private sector.

Additionally, another challenge is overemphasizing the filling of amenity vacancies provided accordingly while neglecting the contextual variance among different neighbourhoods. Previous nationwide practices have exposed the pitfalls of oversimplified top–down and standardised urban renewal strategies in responding to diverse and nonstandardised local needs. To improve residential satisfaction and foster liveable neighbourhoods, various governmental guidelines have emphasized that the revitalization of old neighbourhoods should be made case by case. “One plan for one neighbourhood” should be advocated hereafter. Thus, the IPA–Kano model adopted in this study provides an effective method to determine the local priorities for the improvement in amenities. The model can be applied for prioritizing improvements either for a specific group or an individual neighbourhood.

Lastly, blueprint drawings should be developed together by all stakeholders to visualise the aspired future of neighbourhood development. Public participation is an important method to ensure the local demands can be responded to through urban renewal. However, it has been always criticized that public participation is formalistic and the effect of urban renewal does not substantially meet residents’ aspirations. In this study, 79.3% of the respondents declared that they have never attended any form of meeting conducted by their neighbourhood residential committee. On the governmental side, the responsible department aims to have the renewal plan endorsed by the involved residents as quickly as possible. Therefore, they would rather narrow down the size of public participation to speed up the participation procedure. On the residential side, given that they were not well informed regarding renewal issues, the degree of their participation is very limited. Some residents did not even know that there is a public consultation as part of the whole process. To address this problem, this study advocates that a blueprint plan, which should be mainly drawn by the affected residents, should be used in all urban renewal projects. This might not only ensure that there is a clear and desirable objective to be achieved, but also provide a reference for the evaluation of the project.

## 7. Conclusions

To improve the efficiency of resources utilization and address the contextual variance of local demands among different neighbourhoods, prioritizing the improvement in amenities is critical in navigating neighbourhood revitalization toward a people-centric direction. This study investigated residents’ perceptions of amenity usage and demands in Wuhan through a questionnaire survey and applied the IPA–Kano model to determine the priorities for improving overall neighbourhood satisfaction. The findings suggest that the deficiency of desired amenities is more significant and challenging in old danwei neighbourhoods, and that vulnerable groups should be given special priority because of their higher dependence on neighbourly amenities compared with other groups. Although a similar frequency of amenity usage was found among neighbourhoods, different patterns of significant associations between amenities and neighbourhood satisfaction were found through logistic regression analysis. This supports the rationale of Campbell’s environmental psychology model, which illustrated that the links between objective attributes and neighbourhood satisfaction are intermediated by subjective perception and additionally affected widely by residents’ personal characteristics [2]. This research adopted the IPA–Kano model to provide an adaptive and useful solution for ensuring that the improvements in amenities largely meet the corresponding local demands.

The medium level of the overall mean satisfaction concerning neighbourhood amenities (3.44/5) reveals that there is still room to grow in improving service provisions in Wuhan, particularly for public open spaces, parking lots, garbage collection points, elderly care centres, public toilets, property management centres. The more frequent use by the elderly and their lower satisfaction with elderly care centres, activity squares, reading rooms, open spaces, and garbage collection points imply their higher dependence on and sensitivity to these amenities than other age groups. The challenges derived from an ageing population and the built environment highlight the urgency and importance by elderly residents for improving the desired amenities in old neighbourhoods. Regarding this, the IPA–Kano model identified that the priority of improvements should be given to reading rooms, elderly service stations, property management centres, neighbourhood squares, garbage collection points, and parking lots. Given the limited available unoccupied space in old neighbourhoods, this study advocates for building neighbourhood municipal centres to collectively provide the deficient amenities.

The findings discourage the endeavour of simply adopting standardised and universal approaches in neighbourhood improvement that fits all scenarios of urban decay. As asymmetric influences of various amenities on neighbourhood satisfaction were identified in this research, prioritizing the improvements in amenities should be made case by case to significantly enhance local residents’ neighbourhood satisfaction. This result supports the existing national policy of revitalizing the old neighbourhoods by type and sequence in China. Additionally, the findings expose that public participation during the neighbourhood revitalization process is significantly weak, which intensified the mismatch between supply and demand. Thus, this research proposed a reference for combining the bottom–up and top–down approaches to urban renewal. The transdisciplinary application of IPA–Kano modelling in this study allows authorities and professionals to more effectively utilize available and limited resources to significantly enhance existing residents’ satisfaction [35]. Furthermore, the amenities accommodate healthy factors, such as reading, physical activities, community engagement, and access to health service, which were significantly associated with overall neighbourhood satisfaction. This highlights the crucial role of neighbourhood amenities in developing healthy and liveable cities.

Concerning general situations, a conclusive answer to what specific amenities should be firstly prioritized is not the focus of this study. Instead, this research provides a method to investigate asymmetric influences of various urban factors on overall satisfaction. Therefore, similar studies can be conducted in different scenarios of urban development to further explore the application of the IPA–Kano model in urban studies. Given the importance of equity in developing sustainable cities, future studies should focus on suburban or resettled neighbourhoods in China, where low-income residents generally live.

## Figures and Tables

**Table 1:** Shortlist categories and specific amenities.

No.	Category	Specific Amenities
1	Administrative and Management	Neighbourhood Civil Centres, Service Stations, Offices of Property Owners Committees, Police Stations, Property Management Offices
2	Education	Middle Schools, Elementary Schools, Kindergartens, Other Training Institutes
3	Healthcare	Neighbourhood Hospitals, Clinics, Healthcare Stations, Rehabilitation Centres, Pharmacies
4	Service and Business	Convenience Stores, Fresh Markets, Dining Halls, Elderly Care Centres, Grocery Stores, Bank and ATMs, Delivery Points, Laundries, Bookstores
5	Civic and Transportation	Public Toilets, Elevators and Lifts, Garbage Collection Points, Parking Lots, Transportation Stations, Metro Stations, Shared Bicycle Points
6	Public, Cultural, and Sports	Outdoor Fitness Areas, Fitness Centres, Chess Rooms, Ball Courts and Game Rooms, Elderly Activity Rooms, Neighbourhood Parks or Outside Sitting Areas, Reading Rooms

**Table 2:** Socioeconomic characteristics of all the valid respondents [16].

Category	Group	Frequency	Percentage (%)
Gender	Male	2342	45.9
	Female	2758	54.1
Age	18–25	541	10.6
	26–35	1394	27.3
	36–45	1186	23.3
	46–60	1188	23.3
	60 above	791	15.5
Education	Lower than primary school	1	0.0
	Elementary school	84	1.6
	Secondary school	2468	48.4
	College or higher diploma	2481	48.6
	Postgraduate	66	1.3
Property Rights of the Occupant	Property owner	4592	90.0
	Tenant	508	10.0
Family Structure	Living alone	146	2.9
	Couple only	915	17.9
	Nuclear family	2361	46.3
	Living with parents	632	12.4
	Extended family consisting of three generations	720	14.1
	Other	326	6.4
Hukou Status	Local hukou	4477	87.8
	Nonlocal hukou	623	12.2
Length of Living	Less than one year	46	0.9
	One to three years	611	12.0
	Four to six years	1325	26.0
	Seven to ten years	832	16.3
	Longer than ten years	2286	44.8
Occupation	Civil servant	130	2.5
	Public institution professional	693	13.6
	Private company employee	1280	25.1
	Worker	355	7.0
	Self-employed entrepreneur	397	7.8
	Student	108	2.1
	Freelancer	276	5.4
	Other	667	13.1
Personal Monthly Income	3000 RMB and below	730	14.3
	3000–5000 RMB	2442	47.9
	5001–8000 RMB	1516	29.7
	8001–10,000 RMB	339	6.6
	10,000 RMB and above	73	1.4
Total		5100	
Mean value of overall satisfaction with neighbourhood life		3.44	56.58% of the respondents feel satisfied or highly satisfied

**Table 3:** The ranking of the rate of frequent usage and perceived to-be-improved amenities.

	Order	Elderly Group (60 Years Old or Above)		Young and Middle-Age Group (From 18 to 59 Years Old)	
The ranking of the percentage of respondents who selected the amenity as frequently used ones.	1	Fresh Markets	90.3%	Supermarkets	85.5%
	2	Supermarkets	79.1%	Fresh Markets	79.6%
	3	Restaurants	56.3%	Restaurants	74.6%
	4	Bus Stations	48.4%	Convenience Stores	65.8%
	5	Community Outside Sitting Areas	48.2%	Delivery and Pick-up Stations	60.4%
	6	Pharmacies	46.1%	Bus Stations	59.2%
	7	Convenience Stores	42.6%	Subway Stations	50.0%
The ranking of the amenities to be urgently improved (the percentage of respondents who thought the amenity should be urgently improved).	1	Community Outside Sitting Areas	30.0%	Community Outside Sitting Areas	25.5%
	2	Fitness Areas	29.3%	Parking Lots	23.4%
	3	Elderly Activity Centres	24.9%	Fitness Areas	20.8%
	4	Public Toilets	19.8%	Fresh Markets	16.0%
	5	Parking Lots	18.7%	Public Toilets	10.7%
	6	Fresh Markets	13.3%	Garbage Collection Points	10.6%
	7	Community Hospitals	11.5%	Property Management Centres	9.9%

**Table 4:** The ranking of mean value for elderly satisfaction with individual amenities in old danwei neighbourhoods.

	N	Mean Value
Convenience Stores and Supermarkets	482	3.88
2. Dining Halls	482	3.74
3. Pharmacies	482	3.72
4. Transportation Stations	482	3.71
5. Delivery Points	482	3.70
6. Neighbourhood Hospitals and Clinics	482	3.67
7. Elementary and Primary Schools	482	3.62
8. Bank Branch and ATMs	482	3.60
9. Neighbourhood Civil Centres	482	3.58
10. Fresh Markets	482	3.55
11. Kindergartens	482	3.49
12. Other Administrative Facilities	482	3.42
13. Shared Bicycle Points	482	3.34
14. Other Training Institutes	482	3.25
15. Public Toilets	482	3.17
16. Chess and Entertaining Room	482	3.11
17. Elderly Service Stations	482	3.05
18. Garbage Collection Points	482	3.02
19. Neighbourhood Parks and sitting-out Areas	482	2.94
20. Property Management Offices	482	2.92
21. Fitness Rooms or Areas	482	2.92
22. Reading Rooms	482	2.79
23. Parking Lots	482	2.64

**Table 5:** The three factors and performance matrix.

	Good Performance	Moderate Performance	Poor Performance
**Exciting Factors**	Convenience Stores	Fresh Markets, Bank Branches, Kindergartens, Public Toilets, Neighbourhood Service Centre	None
**Important Performance Factors**	Community Hospitals, Delivery and Pick Up Stations, Bus Stations Pharmacies	Privately-Owned Education and Training Centres, Chess Rooms	Property Management Centres, Garbage Collection Points, Neighbourhood Squares, Parking Lots
**Basic Factors**	Restaurants	Other Administrative Facilities, Service Stations	Reading Rooms, Elderly Service Stations

## Data Availability

Some or all data, models, or codes that support the findings of this study are available from the corresponding author upon reasonable request.

## Appendix B. Results of Ordinal Logistical Regression

**Table A1:** Model-fitting information.

Model	−2 Log Likelihood	Chi-Square	df	Sig.
Intercept Only	1103.154			
Final	723.85	379.304	105	0.000

Link function: Cauchit.

**Table A2:** Goodness of fit.

	Chi-Square	df	Sig.
Pearson	2184.723	1799	0.000
Deviance	721.077	1799	1.000

Link function: Cauchit.

**Table A3:** Pseudo R-square.

Cox and Snell	0.545
Nagelkerke	0.606
McFadden	0.343

Link function: Cauchit.

**Table A4:** Parameter estimates.

		Estimate	Std. Error	Wald	df	Sig.	95% Confidence Interval	
							Lower Bound	Upper Bound
Threshold	(overall neighbourhood satisfaction =1)	−85.656	60.675	1.993	1	0.158	−204.577	33.266
	(overall neighbourhood satisfaction = 2)	−7.989	12.309	0.421	1	0.516	−32.115	16.137
	(overall neighbourhood satisfaction = 3)	−0.573	12.316	0.002	1	0.963	−24.713	23.566
	(overall neighbourhood satisfaction = 4)	10.052	12.216	0.677	1	0.411	−13.892	33.995
Location	[Convenience Stores = 2]	−1.548	1.626	0.906	1	0.341	−4.734	1.639
	[Convenience Stores = 3]	−4.268	1.419	9.047	1	0.003	−7.049	−1.487
	[Convenience Stores = 4]	−1.961	1.158	2.870	1	0.090	−4.230	0.308
	[Convenience Stores = 5]	0 ^a^	.	.	0	.	.	.
	[Fresh Markets = 1]	3.968	2.075	3.657	1	0.056	−0.099	8.034
	[Fresh Markets = 2]	2.717	1.011	7.221	1	0.007	0.735	4.699
	[Fresh Markets = 3]	3.181	1.011	9.898	1	0.002	1.199	5.162
	[Fresh Markets = 4]	2.521	0.869	8.410	1	0.004	0.817	4.224
	[Fresh Markets = 5]	0 ^a^	.	.	0	.	.	.
	[Dining Halls = 2]	−1.220	1.128	1.170	1	0.279	−3.432	0.991
	[Dining Halls = 3]	−1.448	0.939	2.378	1	0.123	−3.288	0.392
	[Dining Halls = 4]	−1.367	0.821	2.772	1	0.096	−2.976	0.242
	[Dining Halls = 5]	0 ^a^	.	.	0	.	.	.
	[Elderly Care Centres = 1]	−7.037	2.308	9.296	1	0.002	−11.560	−2.513
	[Elderly Care Centres = 2]	−3.096	1.788	2.0998	1	0.083	−6.601	0.408
	[Elderly Care Centres = 3]	−0.657	1.710	0.147	1	0.701	−4.009	2.695
	[Elderly Care Centres = 4]	−0.724	1.670	0.188	1	0.665	−3.998	2.550
	[Elderly Care Centres = 5]	0 ^a^	.	.	0	.	.	.
	[Bank and ATMs = 1]	−1.020	8.681	0.014	1	0.907	−18.035	15.996
	[Bank and ATMs = 2]	3.569	2.025	3.107	1	0.078	−0.399	7.537
	[Bank and ATMs = 3]	4.026	1.997	4.063	1	0.044	0.111	7.941
	[Bank and ATMs = 4]	5.379	1.996	7.261	1	0.007	1.466	9.292
	[Bank and ATMs = 5]	0 ^a^	.	.	0	.	.	.
	[Delivery Points = 1]	0.037	11.639	.000	1	0.997	−22.775	22.849
	[Delivery Points = 2]	−0.281	1.909	.022	1	0.883	−4.022	3.460
	[Delivery Points = 3]	−2.875	1.432	4.031	1	0.045	−5.682	−0.068
	[Delivery Points = 4]	−1.436	1.281	1.256	1	0.262	−3.946	1.075
	[Delivery Points = 5]	0 ^a^	.	.	0	.	.	.
	[Middle and Elementary Schools = 2]	−1.750	2.514	0.485	1	0.486	−6.679	3.178
	[Middle and Elementary Schools = 3]	−1.324	2.384	0.308	1	0.579	−5.997	3.349
	[Middle and Elementary Schools = 4]	−2.311	2.368	0.953	1	0.329	−6.952	2.329
	[Middle and Elementary Schools = 5]	0 ^a^	.	.	0	.	.	.
	[Kindergartens = 1]	−3.035	3.085	0.968	1	0.325	−9.081	3.011
	[Kindergartens = 2]	0.251	1.670	0.023	1	0.880	−3.021	3.524
	[Kindergartens = 3]	0.491	1.640	0.090	1	0.765	−2.723	3.706
	[Kindergartens = 4]	−0.776	1.578	0.242	1	0.623	−3.868	2.317
	[Kindergartens = 5]	0 ^a^	.	.	0	.	.	.
	[Other Training Institutes = 1]	−42.234	199.530	.045	1	0.832	−433.306	348.838
	[Other Training Institutes = 2]	6.788	4.474	2.302	1	0.129	−1.981	15.557
	[Other Training Institutes = 3]	5.280	4.411	1.433	1	0.231	−3.366	13.927
	[Other Training Institutes = 4]	6.226	4.476	1.935	1	0.164	−2.547	14.998
	[Other Training Institutes = 5]	0 ^a^	.	.	0	.	.	.
	[Neighbourhood Hospitals and Clinics = 1]	8.591	3.394	6.407	1	0.011	1.939	15.243
	[Neighbourhood Hospitals and Clinics = 2]	0.159	1.105	0.021	1	0.886	−2.007	2.324
	[Neighbourhood Hospitals and Clinics = 3]	2.396	1.053	5.176	1	0.023	0.332	4.459
	[Neighbourhood Hospitals and Clinics = 4]	0.673	0.924	0.531	1	0.466	−1.138	2.483
	[Neighbourhood Hospitals and Clinics = 5]	0 ^a^	.	.	0	.	.	.
	[Pharmacies = 1]	−5.362	3.336	2.584	1	0.108	−11.900	1.176
	[Pharmacies = 2]	−7.030	1.529	21.143	1	0.000	−10.026	−4.033
	[Pharmacies = 3]	−5.379	1.314	16.751	1	0.000	−7.955	−2.803
	[Pharmacies = 4]	−5.531	1.236	20.021	1	0.000	−7.953	−3.108
	[Pharmacies = 5]	0 ^a^	.	.	0	.	.	.
	[Neighbourhood Civil Centres = 1]	−4.404	3.097	2.021	1	.155	−10.475	1.667
	[Neighbourhood Civil Centres = 2]	−4.686	1.842	6.475	1	0.011	−8.295	−1.077
	[Neighbourhood Civil Centres = 3]	−2.910	1.683	2.989	1	0.084	−6.209	0.389
	[Neighbourhood Civil Centres = 4]	−1.761	1.610	1.196	1	0.274	−4.917	1.395
	[Neighbourhood Civil Centres = 5]	0 ^a^	.	.	0	.	.	.
	[The Offices of Property Owners Committees and Property Management = 1]	2.730	1.833	2.217	1	0.136	−0.863	6.324
	[The Offices of Property Owners Committees and Property Management = 2]	1.514	1.469	1.061	1	0.303	−1.366	4.394
	[The Offices of Property Owners Committees and Property Management = 3]	3.716	1.449	6.578	1	0.010	0.876	6.556
	[The Offices of Property Owners Committees and Property Management = 4]	4.230	1.427	8.783	1	0.003	1.433	7.028
	[The Offices of Property Owners Committees and Property Management = 5]	0 ^a^	.	.	0	.	.	.
	[Other Administrative facilities = 1]	−3.916	4.117	0.905	1	0.342	−11.986	4.154
	[Other Administrative facilities = 2]	3.240	2.370	1.869	1	0.172	−1.405	7.885
	[Other Administrative facilities = 3]	−0.508	2.130	0.057	1	0.812	−4.682	3.666
	[Other Administrative facilities = 4]	−0.131	2.100	0.004	1	0.950	−4.246	3.984
	[Other Administrative facilities = 5]	0 ^a^	.	.	0	.	.	.
	[Outdoor and indoor fitness areas = 1]	−5.275	3.144	2.816	1	0.093	−11.436	0.886
	[Outdoor and indoor fitness areas = 2]	−4.341	1.929	5.065	1	0.024	−8.122	−0.561
	[Outdoor and indoor fitness areas = 3]	−4.315	1.888	5.224	1	0.022	−8.016	−0.615
	[Outdoor and indoor fitness areas = 4]	−3.237	1.777	3.318	1	0.069	−6.719	0.246
	[Outdoor and indoor fitness areas = 5]	0 ^a^	.	.	0	.	.	.
	[Entertainment Room = 1]	−3.474	2.563	1.836	1	0.175	−8.498	1.551
	[Entertainment Room = 2]	−2.505	1.390	3.249	1	0.071	−5.229	0.219
	[Entertainment Room = 3]	−2.343	1.355	2.992	1	0.084	−4.998	0.312
	[Entertainment Room = 4]	−1.728	1.279	1.824	1	0.177	−4.234	0.779
	[Entertainment Room = 5]	0 ^a^	.	.	0	.	.	.
	[Reading Rooms = 1]	2.675	1.924	1.934	1	0.164	−1.096	6.447
	[Reading Rooms = 2]	2.202	1.420	2.404	1	0.121	−0.582	4.985
	[Reading Rooms = 3]	0.714	1.369	0.272	1	0.602	−1.969	3.396
	[Reading Rooms = 4]	1.058	1.397	0.573	1	0.449	−1.681	3.797
	[Reading Rooms = 5]	0 ^a^	.	.	0	.	.	.
	[Neighbourhood Parks or Outside Sitting Areas = 1]	−3.038	2.476	1.505	1	0.220	−7.892	1.816
	[Neighbourhood Parks or Outside Sitting Areas = 2]	0.114	1.254	0.008	1	0.927	−2.343	2.572
	[Neighbourhood Parks or Outside Sitting Areas = 3]	−0.392	1.203	0.106	1	0.744	−2.750	1.966
	[Neighbourhood Parks or Outside Sitting Areas = 4]	1.263	1.156	1.192	1	0.275	−1.004	3.529
	[Neighbourhood Parks or Outside Sitting Areas = 5]	0 ^a^	.	.	0	.	.	.
	[Squares = 1]	−5.162	2.188	5.564	1	0.018	−9.451	−0.873
	[Squares = 2]	0.458	0.527	0.756	1	0.385	−0.574	1.490
	[Squares = 3]	2.333	1.106	4.448	1	0.035	0.165	4.501
	[Squares = 4]	1.732	0.520	11.083	1	0.001	0.712	2.751
	[Squares = 5]	0 ^a^	.	.	0	.	.	.
	[Garbage Collection Points = 1]	2.539	2.251	1.272	1	0.259	−1.874	6.952
	[Garbage Collection Points = 2]	−0.704	1.501	0.220	1	0.639	−3.646	2.239
	[Garbage Collection Points = 3]	0.463	1.518	0.093	1	0.761	−2.513	3.438
	[Garbage Collection Points = 4]	1.781	1.461	1.485	1	0.223	−1.083	4.645
	[Garbage Collection Points = 5]	0 ^a^	.	.	0	.	.	.
	[Parking Lots = 1]	−6.206	2.750	5.092	1	0.024	−11.595	−0.816
	[Parking Lots = 2]	−3.112	2.610	1.422	1	0.233	−8.227	2.002
	[Parking Lots = 3]	−2.567	2.600	0.974	1	0.324	−7.663	2.530
	[Parking Lots = 4]	−4.994	2.677	3.479	1	0.062	−10.241	0.254
	[Parking Lots = 5]	0 ^a^	.	.	0	.	.	.
	[Transportation Stations = 1]	−4.509	2.111	4.564	1	0.033	−8.646	−0.372
	[Transportation Stations = 2]	−1.556	1.603	0.942	1	0.332	−4.698	1.586
	[Transportation Stations = 3]	−4.477	1.580	8.028	1	0.005	−7.574	−1.380
	[Transportation Stations = 4]	−2.777	1.483	3.505	1	0.061	−5.684	0.130
	[Transportation Stations = 5]	0 ^a^	.	.	0	.	.	.
	[Shared Bicycle Points = 1]	−1.504	6.592	0.052	1	0.820	−14.424	11.416
	[Shared Bicycle Points = 2]	0.925	5.144	0.032	1	0.857	−9.156	11.007
	[Shared Bicycle Points = 3]	0.270	5.109	0.003	1	0.958	−9.744	10.284
	[Shared Bicycle Points = 4]	0.297	5.104	0.003	1	0.954	−9.707	10.300
	[Shared Bicycle Points = 5]	0 ^a^	.	.	0	.	.	.
	[Gender = 1]	−1.482	0.430	11.851	1	0.001	−2.325	−0.638
	[Gender = 2]	0 ^a^	.	.	0	.	.	.
	[Education= 2]	−0.264	1.057	0.063	1	0.802	−2.335	1.807
	[Education = 3]	0.028	0.856	0.001	1	0.974	−1.650	1.706
	[Education = 4]	0 ^a^	.	.	0	.	.	.
	[Family Structure = 1]	0.631	1.406	0.201	1	0.654	−2.124	3.386
	[Family Structure = 2]	0 ^a^	.	.	0	.	.	.
	[family structure = 1]	−1.661	1.384	1.441	1	0.230	−4.373	1.051
	[family structure = 2]	−2.731	1.253	4.755	1	0.029	−5.187	−0.276
	[family structure = 3]	−1.516	1.766	0.738	1	0.390	−4.977	1.944
	[family structure = 4]	−4.968	2.174	5.221	1	0.022	−9.230	−0.706
	[family structure = 5]	−2.288	1.244	3.382	1	0.066	−4.727	0.150
	[family structure = 6]	0 ^a^	.	.	0	.	.	.
	[Hukou Status = 1]	1.763	1.303	1.830	1	0.176	−0.791	4.318
	[Hukou Status = 2]	0 ^a^	.	.	0	.	.	.
	[Length of Living = 2]	0.294	1.624	0.033	1	0.857	−2.889	3.476
	[Length of Living = 3]	0.026	1.183	0.000	1	0.982	−2.291	2.344
	[Length of Living = 4]	0.128	0.641	0.040	1	0.842	−1.129	1.384
	[Length of Living = 5]	0 ^a^	.	.	0	.	.	.
	[Personal Monthly Income = 1]	−0.976	9.188	0.011	1	0.915	−18.984	17.031
	[Personal Monthly Income = 2]	−0.300	9.184	0.001	1	0.974	−18.301	17.700
	[Personal Monthly Income = 3]	0.718	9.203	0.006	1	0.938	−17.319	18.755
	[Personal Monthly Income = 4]	0 ^a^	.	.	0	.	.	.

Link function: Cauchit. ^a^. This parameter is set to zero because it is redundant.

**Table A5:** Test of parallel lines ^a^.

Model	−2 Log Likelihood	Chi-Square	df	Sig.
Null Hypothesis	723.850			
General	579.806 ^b^	144.044 ^c^	315	1.000

The null hypothesis states that the location parameters (slope coefficients) are the same across response categories. ^a^. Link function: Cauchit. ^b^. The log-likelihood value cannot be further increased after maximum number of step-halving. ^c^. The Chi-square statistic is computed based on the log-likelihood value of the last iteration of the general model. Validity of the test is uncertain.
